# Characterizing functional DNA damage and response caused by the combination of CHK1 and WEE1 inhibitors in ovarian and breast cancer models

**DOI:** 10.1038/s44276-024-00048-8

**Published:** 2024-04-03

**Authors:** Adam Stewart, Jiin Song, Lisa Pickard, Giovanna Muggiolu, Sylvie Sauvaigo, Alexis De Haven Brandon, Florence Raynaud, Udai Banerji

**Affiliations:** 1 The Institute of Cancer Research, London, UK; 2 LXRepair, Grenoble, France

## Abstract

**Background:**

We proposed to quantify reduction of functional DNA damage response (DDR) mechanisms caused by the combination of CHK1 and WEE1 inhibitors.

**Methods:**

Survival of cells and tumor growth in-vitro and in-vivo caused by the combination of the CHK1 inhibitor SRA737 and the WEE1 inhibitor adavosertib was studied in OVCAR3 and MDA-MB 436 cells. Functional DNA damage was quantified using in vitro cell free DNA assays.

**Results:**

The combination of SRA737 and adavosertib caused significant reduction of survival of cells and DNA damage in-vitro and growth inhibition in-vivo. Studies using functional DDR assays found significant changes in the functional capacity of OVCAR3 but not MDA-MB 436 cells to repair DNA damage using multiple mechanisms including intra strand cross link repair, nucleotide excision repair, homologous recombination and non-homologous end joining. This study, for the first time provides a mechanistic insight into differences in the reduction in functional capacity of cells to repair DNA when exposed to CHK1 and WEE1 inhibitors.

**Conclusion:**

The combination of the CHK1 inhibitor SRA737 and WEE1 inhibitor adavosertib causes growth inhibition in-vitro and in-vivo, but differential functional inhibition of DDR in the models studied.

## Introduction

Multiple orthogonal experimental techniques point to the synergy in growth inhibition between CHK1 and WEE1 inhibitors. Synthetic lethal SiRNA screens have identified CHK1 deletion to be synthetically lethal in leukemic cell lines exposed to WEE1 inhibitors [1], while SiRNA screens of ovarian cancer cells exposed to CHK1 inhibitors have identified deletion of WEE1 to be synthetically lethal [2]. Further, studies of synthetically lethal effects of drugs with WEE1 inhibitors revealed CHK1 inhibitors as a hit [3]. Unbiased screens of drugs in breast, colon, pancreatic and lung cancers have also shown synergy in CHK1 and WEE1 inhibitors across multiple cell lines [4, 5].

Both CHK1 and WEE1 are key to the fidelity of G2/M checkpoint and inhibition of CHK1 and WEE1 leads to the abrogation of the G2/M checkpoint. Combinations of these agents with chemotherapy have hypothesized replication stress caused by the chemotherapeutic agent and cell death due to mitotic catastrophe due to abrogation of the G2/M checkpoint [6, 7]. These concepts have been explored in the clinical setting [8, 9]. Combination of CHK1 and WEE1 inhibitor have also been shown to be synergistic but interestingly, multiple investigators have found cells accumulating in S phase when treated with the combination [2, 3, 10]. Further studies have shown mechanism of the synergy of S phase DNA damage due to the combination of CHK1 and WEE1 inhibitors is due to be unscheduled initiation of replication [10].

We aimed to study for the first time, effects of the combination of CHK1 and WEE1 inhibitors on functional capacity of DNA damage response (DDR) using cell free assays [11]. We used two well characterized clinical compounds SRA737 (CHK1 inhibitor) [7, 9, 12] and adavosertib [13, 14] (WEE1 inhibitor) in OVCAR3 (cyclin E overexpressing) and MDA-MB-436 (*BRCA1* mutated) cell line models.

## Materials and methods

### Cell lines, drugs and culture medium

Cell lines OVCAR3 and MDA-MB-436 was purchased from ATCC. The cells were grown in RPMI-1640 (11835–063, Gibco). Cells were incubated at 37^o^C with 5% CO2. All cell lines used in experiments were between 4 and 28 passages. Cell lines were tested for Mycoplasma using MycoAlert (LT-07–218, Lonza) within 3 weeks before use. SRA737 was kindly provided by Sierra Oncology and adavosertib was provided by Astra Zeneca.

#### Clonogenic assays

Colony formation in OVCAR-3 and MDA-MB-436 lines was assessed via clonogenic assays in 12 well plates (3513, Corning Inc). Seeding density was first optimized. Cells were allowed to adhere for 24 hours before dosing. Initially plates dosed with a serial dilution of either Adavosertib or SRA737 and incubated for 14 days. Plates stained with 0.5% crystal violet solution (61135, Sigma Aldrich). Colony numbers per well were enumerated via the GelCount system (Oxford Optronix, software version 1.2.1.0). Survival fraction 50 (SF_50_) was defined as concentration of drug at which caused 50% of the survival fraction caused by the DMSO control was calculated from a 9 concentration dose response curve using GraphPad Prism (v8.01, GraphPad Software LLC). The experiments were carried in triplicate as independent biological repeats.

### Cell cycle analysis

OVCAR-3 and MDA-MB-436 cells were exposed to either 1xSF_50_ and incubated for either 24 hours. Post drug exposed media collected to retain dead cells, which were spiked back in. Cells were detached via trypsinisation, pelleted and fixed in cold 100% ethanol. Cells left to fix overnight at 4^o^C. Cells subsequently washed and resuspend in 500 µl PBS, 60 µl RNase A (Roche, 10109142002) and 40 µl propidium iodide (Sigma-Aldrich, P4864). The cell suspension was left at 4^o^C in the dark for at least 30 minutes. DNA content per cell (via propidium iodide intensity) measured via BD FACsymphony flow cytometer (BD biosciences,) at a wavelength of 561 nm and emission of 610/20. The cell cycle phase was labeled manually and analyzed via FlowJo software (BD biosciences) and graphs were generated using Prism software (GraphPad). The experiments were carried in triplicate as independent biological repeats.

#### Western blot

OVCAR-3 and MDA-MB-436 cells were exposed to SF_50_ concentrations of adavosertib, SRA737 or the combination of adovasertib and SRA737 for 24 hours. Experiments for western blots were carried as a single experiment. Cell pellets were thawed on ice, lysed with 1x RIPA buffer containing PhosSTOP (1:50) and protease inhibitor (1:100) (R0278, 4906845001, I3786 respectively, Sigma) for 30–45 minutes (on ice) before being spun. Protein was quantified via BCA assay (23225 and 23227, Thermo Scientific). 30 µg/µl of protein per well loaded onto 4–12% Bis-Tris (NP0323, Life Technology) gels using MOPS running buffer (NP0001, Life Technology). Gels transferred onto a PVDF membrane (IB24001, Invitrogen) using an IBlot2 (Invitrogen) dry blot system. Membrane incubated over night with primary antibody. Primary antibodies used were p-CHK1Ser345 (CST2348, 1:1000), p-CHK1Ser296 (CST2349 1:1000), p-CDC2 TY15 (1:1000 CST4539), p-H2AX (CST9718, 1:1000), GAPDH (CST5174, 1:10000) from Cell Signaling Technology, Liden Netherlands and cleaved PARP (ab32064, 1:1000) from Abcam, Cambridge, UK. Membranes were imaged on the Li-Cor Odyssey FC system.

#### In vivo studies

90 NSG mice were injected with 5 × 10^6^ OVCAR3 and MDA-MB436 cells subcutaneously in one flank (45 mice per experiment). Dosing and measurements commenced 6 weeks post inoculation. Mice were randomized to 10 per group in each experiment. Group 1-Vehicle for SRA737 QD and vehicle for adavosertib BD 2 consecutive days/7 ×3 weeks); Group 2- adavosertib 75 mg/kg, BD 2 consecutive days/7 ×3 weeks; Group 3- SRA737 120 mg/kg, 2 consecutive days/7 ×3 weeks; Group 4- adavosertib 75 mg BD, 2 consecutive days/7 ×3 weeks and SRA737 120 mg/kg QD 2 consecutive days/7 ×3 weeks (both drugs given on the same days). Tumor measurements and mouse weight were assessed every 3 days. Tumor volumes were assessed as (4/3)πr^3^. Difference between tumor volumes were compared using unpaired t tests. Tumor growth inhibition (%TGI = (1-(Tt/T0)/ (Ct/C0)) / (1-(C0/Ct)) X 100) calculated at Day 28. Tt = TREATED Median volume at end point (28 days since first dose).

T0 = Treated median volume on day 0 (the day we first measure and treatment begins).

Ct = Control median volume at end point (28 days since start of dosing).

C0 = Control median volume on day 0 (the day we first measure and treatment begins).

#### DNA repair testing

##### ExSy-SPOT assay

Cell nuclear lysates were prepared as previously described in [15, 16]. In short, cell pellets were suspended in a hypotonic buffer (1 mL/2 million cells) to destroy the cytoplasmic membrane (10 mM HEPES-KOH, 10 mM KCl, 1.5 mM MgCl_2_, 0.5 mM DTT, 103 µM PMSF, 0.02% Triton X-100) for 20 min on ice. Nuclei were recovered by centrifugation at 2.300 g and suspended in a hypertonic buffer (20 µL/2 million cells ; 10 mM HEPES-KOH, 0.2 mM EDTA-NaOH, 400 mM KCl, 1.5 mM MgCl_2_, 0.5 mM DTT, 103 µM PMSF, 0.7X protease inhibitor, 25% glycerol). Two freezing and thawing cycles were performed to disrupt the nuclear membrane. The nuclear lysates were cleared by a final centrifugation at 16.000 g for 10 minutes. Quantification of protein concentration in the lysates was performed using the MicroBC Assay (Interchim). Excision-synthesis repair capacities were determined using the ExSy-SPOT assay [15–17]. This in-vitro cell free DNA repair assay used double-strand plasmid DNA, containing specific DNA lesions and immobilized at pre-determined sites on a biochip to serve as substrates for the repair reactions for the proteins contained in the lysates. Control non modified plasmid was also immobilized. Extent of repair was tracked by the incorporation of labeled dNTP upon synthesis of the neo DNA strand, by sample polymerases, after lesions removal. Repair activities belonging to base excision repair (BER), nucleotide excision repair (NER) and intra-cross link repair (ICLR) were quantified through repair of 8oxoG (BER), abasic sites (BER), ethenobases (BER), glycols (BER), photoproducts or pyrimidine dimers and 6-4 photoproducts (CPD64) (NER) and cisplatin adducts (ICLR and NER). Standard 50 µL repair assay mix contained 40 µL of ATG buffer (55 mM Hepes KOH pH 7.8, 8.75 mM MgCl_2_, 2.5 mM EDTA, 0.625 mM DTT, 4.25% glycerol, 0.30 mM dATP, 0.30 mM dTTP, 0.30 mM dGTP, 12.50 mM phosphocreatine, 0.0625 mg/mL creatine phosphokinase, 0.125 mg/mL BSA, 1 mM ATP, and 0.30 mM dCTP-Cy3) and 10 µL of the lysate at a final protein concentration of 0.2 mg/mL. Adhesive microarray chambers, forming 21 wells (Grace Bio-Labs) were set on each slide and filled with 20 µL of the repair mix. The repair reaction was run at 30 °C for 3 h. Then the slides were washed in a slide holder for 2 ×3 min in MilliQ water. Water was removed from the slides by 5 min centrifugation at 2500 rpm. The slides were then dried for 15 min at 37◦C. Each sample was analyzed on 2 biochips (technical replicates).

### Next-SPOT

This assay quantifies the balance between several main double strand break (DSB) repair activities [11]. Double strand supercoiled plasmid DNA (SC-plasmid) and AflIII restriction enzyme digested plasmid (Lin-plasmid 4 bases overhang; 1 site per plasmid) were immobilized on a biochip and served as a substrate for different repair reactions leading to the incorporation of Cy3-labeled Lin-plasmid and biotin-labeled dNTP, supplied in the repair reaction, on each immobilized substrate. The immobilized SC-plasmid served as a substrate for homologous recombination (HR) and single strand annealing (SSA)/synthesis dependent strand annealing (SDSA) reactions ; the Lin-plasmid serves as a substrate for non-homologous end joining (NHEJ) and alternate end joining (Alt-EJ) reactions. Ten µL of cell lysates were diluted in 40 µL of the repair buffer (25 mM Tris-HCl pH 7.5, 12.5 mM MgCl_2_, 2.5 mM DTT, 1.875% glycerol, 0.30 mM dTTP, 0.30 mM dGTP, 12.50 mM phosphocreatine, 0.0625 mg/mL creatine phosphokinase, 0.125 mg/mL BSA, 1 mM ATP, 0.30 mM biotin-dCTP, and 2.5 µg/mL of Cy3-Lin plasmid). The repair reaction was carried out for 1 hour at 30 °C. The slide was then rinsed twice with Milli-Q water. The biotin-dCTP was subsequently revealed by a 30 min incubation in a streptavidin-Cy5 solution (0.1 µg/mL) at 30 °C. After 2 washes in Milli-Q water, the slides were dried out. Each sample was analyzed at 2 final protein concentrations (0.1 and 0.2 mg/mL), with technical replicates (2 per condition).

### Fluorescence quantification and data analysis

Fluorescent signals were measured using a microarray scanner (Innoscan 710AL from Innopsys, and the Mapix software). Images were acquired at one wavelength (532 nm (Cy3) for ExSy-SPOT) or two wavelengths (532 nm (Cy3) and 635 nm (Cy5) for NEXT-SPOT). The total fluorescence intensity (FI) of each spot was quantified. Then the normalized fluorescence (NormalizeIt software for ExSy-SPOT (8 spots per condition)) or the mean of the replicates (4 spots per condition for NEXT-SPOT) was calculated for each fluorophore and for each plasmid on the slide. Results were expressed as Fluorescence Intensity (Arbitrary Units). Excision/synthesis repair capacities, obtained with ExSy-SPOT were expressed for each repair pathway analyzed, represented by the repaired lesions (8oxoG, AbaS (abasic sites), Glycols, Etheno (ethenobases), all repaired by BER, CPD-64 (photoproducts) repaired by NER and CisP (cisplatin adducts) repaired by ICLR). DSB repair capacities were for each sample were characterized by 4 values, called HR, NHEJ, SSA and alt-EJ, for each protein concentration tested.

### Statistical analysis

Differences between survival fractions in clonogenic experiments and cells in S phase in cell cycle experiments between controls and cells exposed to the combination of SRA737 and adavosertib in both cell lines was carried out by a paired t test. In the xenograft experiments Difference between tumor volumes were compared using unpaired t tests. While determining statistical significance in between control and treated in different components of the DNA damage repair mechanisms in the ExSy and Next-SPOT was carried out using a paired t test.

## Results

### Survival fraction and xenograft growth inhibition caused by SRA737 and adavosertib on OVCAR3 and MDA-MB- 436 models

**Fig. 1:**
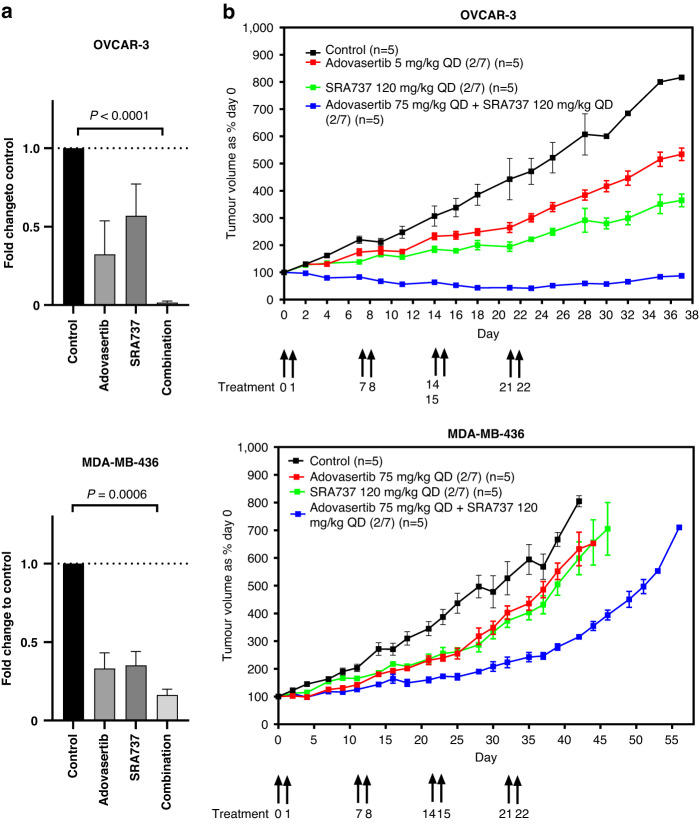
Growth inhibition in ovarian cancer cell lines by SRA737 and adavosertib in-vitro and in-vivo. **a** Colony formation assays when cells were exposed to 1xSF_50_, adavosertib and SRA737 as single agents and in combination for 14 days, *n* = 3. There was significant reduction in colony formation of the samples exposed to combination compared to control in OVCAR3 and MDA-MB-436 cell lines *p* = <0.0001 and *p* = 0.0006 respectively. **b** In vivo evaluation of the combination of adavosertib and SRA737. Mice were treated with oral doses of adavosertib 75 mg/kg BD and SRA737 120 mg/kg QD on day 0,1,7,8,14,15,21 and 22. In the OVCAR-3 xenograft model There was a statistically significant difference in tumor volumes between the control arm and combination therapy arm *p* < 0.0001 on day 28. In the MDA-MB-436 xenograft model, there was a statistically significant difference in tumor volumes between the control arm and the combination therapy arm *p* = 0.0025 on day 28.

We determined the survival fraction (SF_50_) concentrations of SRA737 and adavosertib in OVCAR3 (730 nM and 182 nM, respectively) and MDA-MB-436 cell lines (1819 nM and 546 nM respectively). We then exposed OVCAR3 and MDA-MB-436 cells to single concentrations of SRA737, adovasertib and the combination of SRA737 and adovasertib to their respective SF_50_ concentrations stated above and quantified number of colonies. The combination of SRA737 and adavosertib caused significant reduction of number of colonies compared to control when exposed to SF_50_ concentrations in OVCAR3 and MDA-MB-436 cell lines, *p* < 0.0001 and *p* = 0.0006, respectively (Fig. 1a). When OVCAR3 and MDA-MB-436 were exposed to SF_50_ concentrations of SRA737, adovasertib and the combination of both drugs for 24 hours, it was not possible to measure the proximal mechanism of action of SRA737 in OVCAR3 cells as it was not possible to detect p-Ser296 CHK1 in this cell line, however it was possible to demonstrate target inhibition by SRA737 in MDA-MB-436 as evidenced by reduction in p-Ser296 CHK1. It was possible to demonstrate target inhibition of adovasertib in both cell lines exposed to their respective SF_50_ concentrations by demonstrating reduction in p-Y15 CDK1 (Supplementary Figs. 1 and 2). We studied the in-vivo growth inhibitory effects of the combination of SRA737 120 mg/kg and adavosertib 75 mg/kg administer concomitantly two days on 5 days off × 4 weeks. The combination of SRA737 and adavosertib caused tumor regressions and these tumors were significantly smaller when compared to the non-treatment control on day 28, *p* < 0.0001 in the OVCAR3 xenograft model. The same dosing regimen in the MDA-MB-436 xenograft model caused a significant reduction in tumor size in the combination arm compared to the control arm, *p* = 0.0025, but this did not amount to regressions. The TGI of the combination treatment arm compared to the control arm was 107.9% and 44.7% in the OVCAR3 and MDA-MB-436 xenograft models, respectively (Fig. 1b).

### Determination of effects of cell cycle and DNA damage caused by SRA737 and adavosertib on OVCAR3 and MDA-MB 436 models

**Fig. 2:**
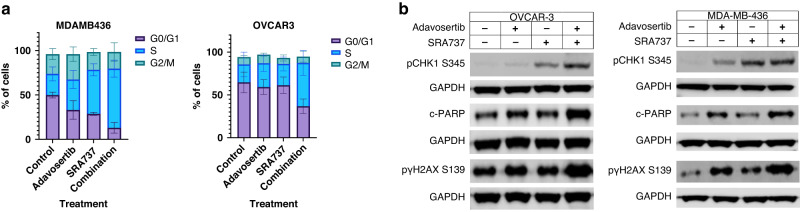
Characterization changes in cell cycle distribution, replication stress, apoptosis and DNA double strand breaks caused by SRA737 and adavosertib. **a** Cell cycle analysis of OVCAR3 and MDA-MB-436 cells exposed to SF_50_ concentrations of SRA737, adavosertib or the combination of SRA737 and adavosertib for 24 h. The experiments were carried in triplicate as independent replicates. Mean values and standard deviations are represented in this figure. **b** OVCAR3 and MDA-MB-436 cells exposed to SF_50_ concentrations of SRA737, adavosertib or the combination of SRA737 and adavosertib for 24 h. Western blot analysis showing an increased levels of pCHK1 Ser345, c-PARP and pγH2AX and in OVCAR3 and MDA-MB436 cell exposed to control, adavosertib, SRA737 and the combination of adavosertib and SRA737 when cells were exposed to SF_50_ concentrations for 24 hours. The experiment was conducted once.

We assessed cell cycle effects of the individual drugs and the combination when OVCAR3 and MDA-MB 436 cells were exposed to SF_50_ concentrations for 24 hours. This showed an increase of cells in S phase caused by the combination compared to control 50.5% Vs 21%, *p* = 0.016 in OVCAR3 66.5% Vs 23.7%, *p* = 0.001 in the MDA-MB 436 cell line (Fig. 2a). We then assessed replication stress, apoptosis and DNA double strand breaks when both cell lines were exposed to SF_50_ concentrations of SRA737 and adavosertib. We demonstrated higher levels of levels of p-CHK1^Ser345^ in addition c-PARP and γH2AX in cells were exposed to the combination of SRA737 and adavosertib compared to either drug alone (Fig. 2b, densitometry in Supplemental Fig. 3).

### Determination functional DNA damage repair caused by SRA737 and adavosertib on OVCAR3 and MDA-MB 436 models

**Fig. 3:**
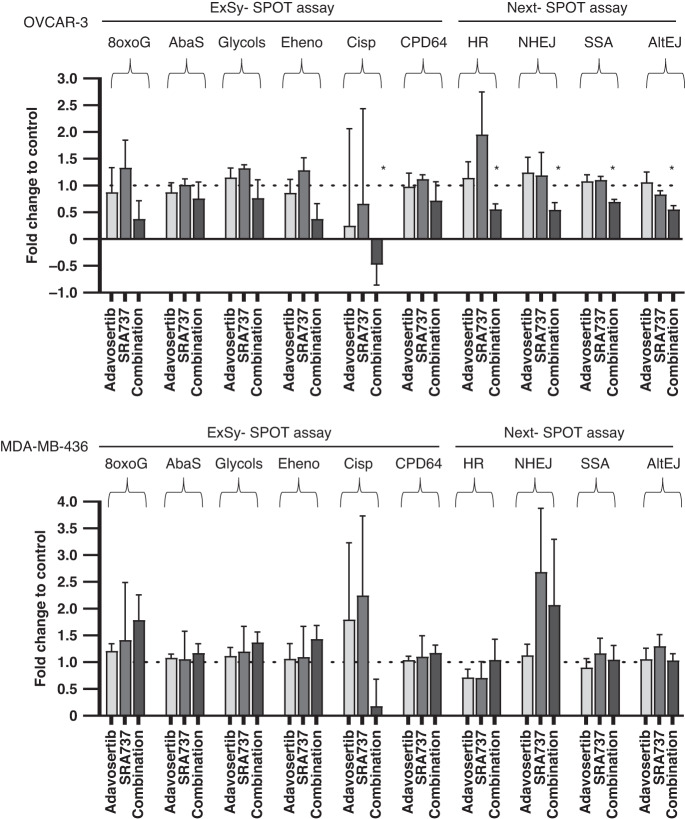
Effects of functional DNA damage repair in cell free assays. Functional capacity of cell lysates to repair DNA damage using the ExSy-SPOT assay; 8oxoG, AbaS, Etheno, Glycols- base excision repair (BER), Cisp- intra strand cross link repair (ISCLR) and Cisp, CPD64-nucleotide excision repair (NER). The Next-SPOT assay studied the ability to repair double strand breaks i.e., homologous recombination (HR), non-homologous end joining (NHEJ), single strand annealing (SSA) and alternative end-joining (ALT-EJ). OVCAR-3 and MDA-MB436 cells were exposed to control, adavosertib and the combination of adavosertib and SRA737. The results have been normalized to the control of each experiment. The experiments were carried out in triplicate and differences between control and treated samples were carried out using pared t tests. Symbol * denotes statistical significance. AbaS Abasic Sites, Etheno = Etheno bases, CPD64 = 6-4 photoproducts, Cisp cisplatin adducts.

OVCAR3 and MDA-MB-436 cell lines were exposed to SF_50_ concentrations of SRA737, adovasertib or the combination for 24 h. Two functional assays were used to further characterize the DDR in these cell lines. The first assay, ExSy-SPOT assay studied the capacity of the cell lysates to perform base excision repair (BER), intra strand cross link repair (ISCLR) and nucleotide excision repair (NER) and the second assay Next-SPOT assay studied the ability to repair double strand breaks i.e., homologous recombination (HR), non-homologous end joining (NHEJ), single strand annealing (SSA) and alternative end-joining (ALT-EJ) [11]. In the OVCAR3 cell line there were no statistically significant differences caused by individual drugs compared to control. However, there was significant difference between control and combination of SRA737 and adavosertib (*p* = 0.022) in one of the measures, “CisP” which measures ISCLR and NER. Further, there was a significant reduction in the ability of the cells repair DNA by HR, NHEJ, SSA and Alt-EJ caused by the combination compared to control (*P* = 0.018, *p* = 0.03, *p* = 0.009 and *p* = 0.009, respectively), (Fig. 3). There were no statistically different changes in the DNA damage response with single agent or combinations in the MDA-MB-436 when compared to control although there was a numerical reduction in the ability to repair DNA by ISCLR and NER. This is for the first time that DNA damage response has been bas been functionally quantified in cells exposed to CHK1 and WEE1 inhibitors.

## Discussion

Previous studies have shown synergistic growth inhibition or cell death resulting from the combination of CHK1 and WEE1 inhibitors [1–5]. Some of these studies have further profiled effects on cell cycle and found accumulation of cells in S phase [3, 10]. The mechanism of cell death and DNA damage caused by this combination are thus different from the traditional use of combinations of CHK1 and WEE1 inhibitors with chemotherapeutic agents such as cisplatin or gemcitabine which causes DNA damage or replication stress and either the CHK1 or WEE1 causing abrogation of G2/M checkpoint resulting in mitotic catastrophe [6, 7]. Groups have tried to understand the mechanism of cell death and have postulated addition of a CHK1 inhibitor to a WEE1 inhibitor causes an increase in unscheduled replication initiation causing DNA damage and accumulation of cells in the S phase [10]. Our experiments have confirmed the effects of the combination on cell survival both in-vitro, accumulation of cells in the S phase and cell death. We have also demonstrated tumor growth inhibition in two xenograft models, suggesting a therapeutic index which could be tested in clinical trials.

We have for the first time demonstrated reduction in the functional ability to repair DNA damage using cell free assays in cancer cells exposed to CHK1 and WEE1 inhibitors. When exposed to the combination of SF_50_ concentrations of SRA737 and adovasertib, we demonstrated accumulation of cells in S phase and cell death in both OVCAR3 and MDA-MB-436 cell lines. Interestingly the functional ability of repair DNA damage was different in the two models. The functional ability of the combination to repair ISCLR, NER,SSA and Alt-EJ was altered in the *BRCA*^WT^ OVCAR3 and not the *BRCA1*^M^ MDA-MB-436 cell line when exposed to the combination SF_50_ concentrations of of SRA737 and adovasertib. However, the OVCAR3 cell line despite not having mutations in *BRCA* genes are known to have functional loss of HR repair possibly due to high non homologous end joining activity [18]. The assays studied shed light on the relative functional capacity of DNA damage response compared to control but bespoke experiments comparing baseline DNA repair capacity of both cell lines grown in a single experiment has not been done. The assay results could be considered a screen of functions of different mechanism of DNA repair and further validation of individual DNA repair functions using orthogonal methods will be needed to confirm these findings. A further hypothesis to explain the differences in the ability to repair DNA between the cell lines is that OVCAR3 is known to have an overexpression of cyclin E which resulting in a rapid transit of cells through the S phase leading to DNA damage in the S phase. OVCAR3 has previously been reported to have overexpression of cyclin E when compared to different ovarian cancer cell lines [19] however in our hands expression of cyclin E, at a protein level was similar in both OVCAR3 and MDA-MB-436 cell lines.

Taken together these experiments show combined effects of a CHK1 and WEE1 inhibitor on clonogenic survival in-vitro and growth inhibition in-vivo in ovarian cancer cell line models. For the first time it provides functional insights into significant reduction in the ability of cells to repair DNA by ICSLR, NER, NEJH, SSA and Alt-EJ caused by the combination in the *BRCA*^WT^ cyclin E overexpressing OVCAR3 ovarian cell line. Clinical trials of both CHK1 [20] and WEE1 [21] inhibitors used as single agents have shown singe agent activity in cyclin E overexpressing ovarian cancer. Our experiments provide novel insights into functional ability of the combination of CHK1 and WEE1 inhibitors to repair DNA and strengthen the case of exploring the combination in cyclin E overexpressing cancers in clinical trials.

## Supplementary information

Supplementary Figure1

Supplementary Figure2

Supplementary Figure3

## Data Availability

The data for this publication will be made available upon review of requests submitted.
