# Supplementary material for: Characterizing functional DNA damage and response caused by the combination of CHK1 and WEE1 inhibitors in ovarian and breast cancer models
Source: BJC Rep. 2024 Apr 3;2:27. doi: 10.1038/s44276-024-00048-8 (PMC11523970; doi:10.1038/s44276-024-00048-8)
Supplement: Supplementary file 1 — Supplementary Figure1 [file 44276_2024_48_MOESM1_ESM.pptx]

## Slide 1
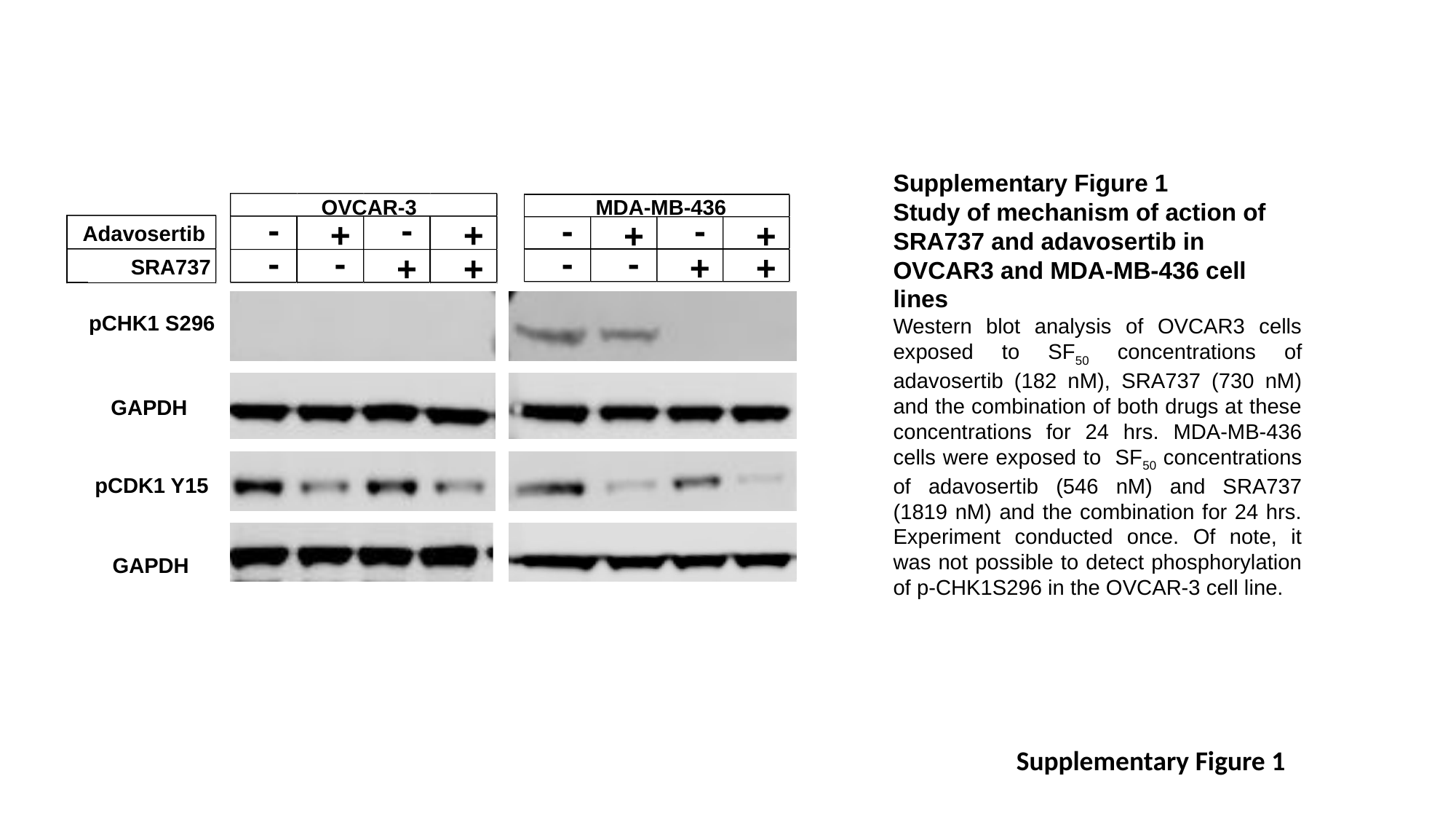

Supplementary Figure 1
Study of mechanism of action of SRA737 and adavosertib in OVCAR3 and MDA-MB-436 cell lines
Western blot analysis of OVCAR3 cells exposed to SF50 concentrations of adavosertib (182 nM), SRA737 (730 nM) and the combination of both drugs at these concentrations for 24 hrs. MDA-MB-436 cells were exposed to SF50 concentrations of adavosertib (546 nM) and SRA737 (1819 nM) and the combination for 24 hrs. Experiment conducted once. Of note, it was not possible to detect phosphorylation of p-CHK1S296 in the OVCAR-3 cell line.
OVCAR-3
-
-
+
+
-
-
+
+
MDA-MB-436
-
-
+
+
-
-
+
+
Adavosertib
SRA737
pCHK1 S296
GAPDH
pCDK1 Y15
GAPDH
Supplementary Figure 1
