# Supplementary material for: Characterizing functional DNA damage and response caused by the combination of CHK1 and WEE1 inhibitors in ovarian and breast cancer models
Source: BJC Rep. 2024 Apr 3;2:27. doi: 10.1038/s44276-024-00048-8 (PMC11523970; doi:10.1038/s44276-024-00048-8)
Supplement: Supplementary file 2 — Supplementary Figure2 [file 44276_2024_48_MOESM2_ESM.pptx]

## Slide 1
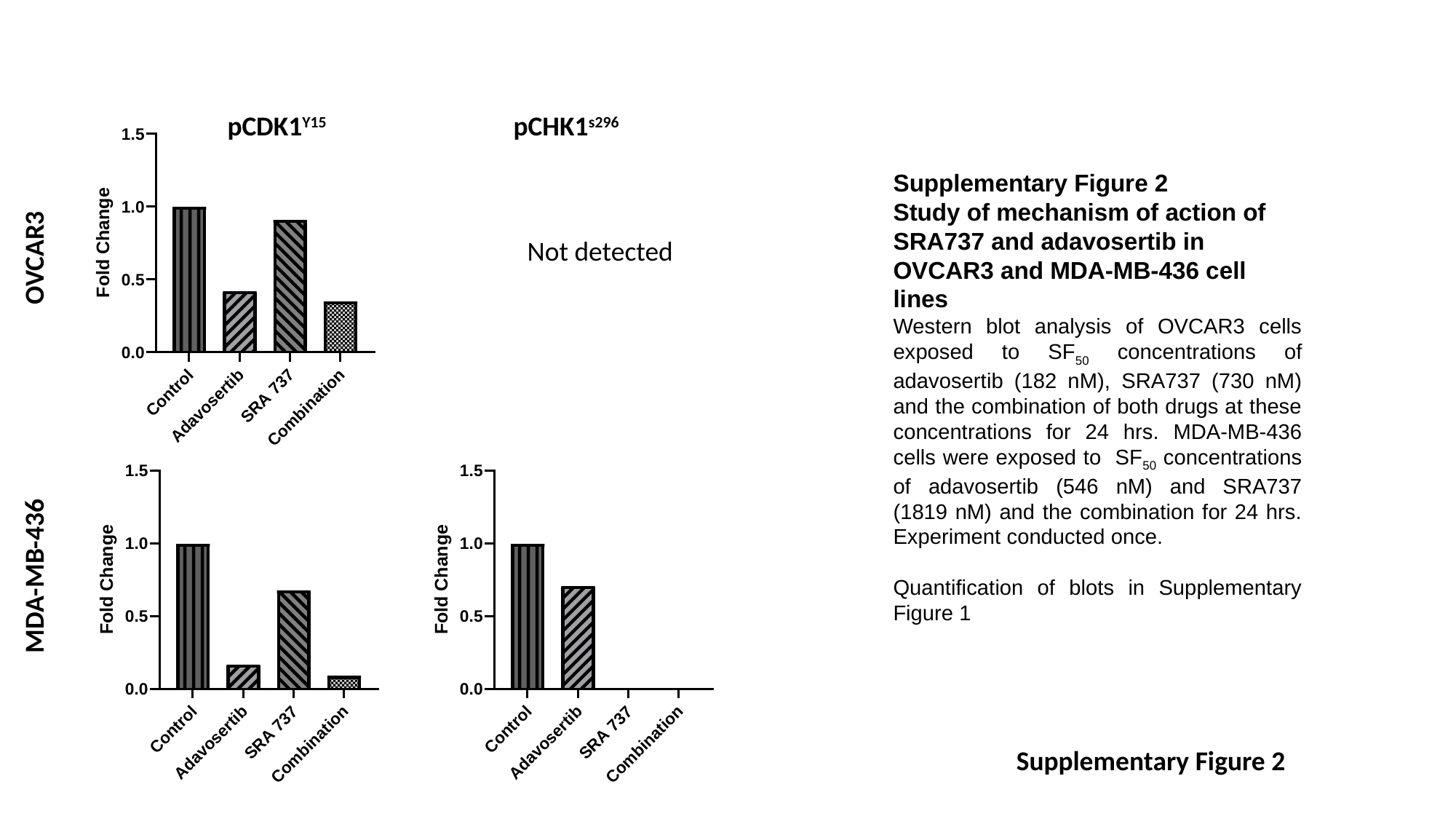

pCDK1Y15		 pCHK1s296
Supplementary Figure 2
Study of mechanism of action of SRA737 and adavosertib in OVCAR3 and MDA-MB-436 cell lines
Western blot analysis of OVCAR3 cells exposed to SF50 concentrations of adavosertib (182 nM), SRA737 (730 nM) and the combination of both drugs at these concentrations for 24 hrs. MDA-MB-436 cells were exposed to SF50 concentrations of adavosertib (546 nM) and SRA737 (1819 nM) and the combination for 24 hrs. Experiment conducted once.
Quantification of blots in Supplementary Figure 1
Not detected
OVCAR3
MDA-MB-436
Supplementary Figure 2
