# Supplementary material for: Characterizing functional DNA damage and response caused by the combination of CHK1 and WEE1 inhibitors in ovarian and breast cancer models
Source: BJC Rep. 2024 Apr 3;2:27. doi: 10.1038/s44276-024-00048-8 (PMC11523970; doi:10.1038/s44276-024-00048-8)
Supplement: Supplementary file 3 — Supplementary Figure3 [file 44276_2024_48_MOESM3_ESM.pptx]

## Slide 1
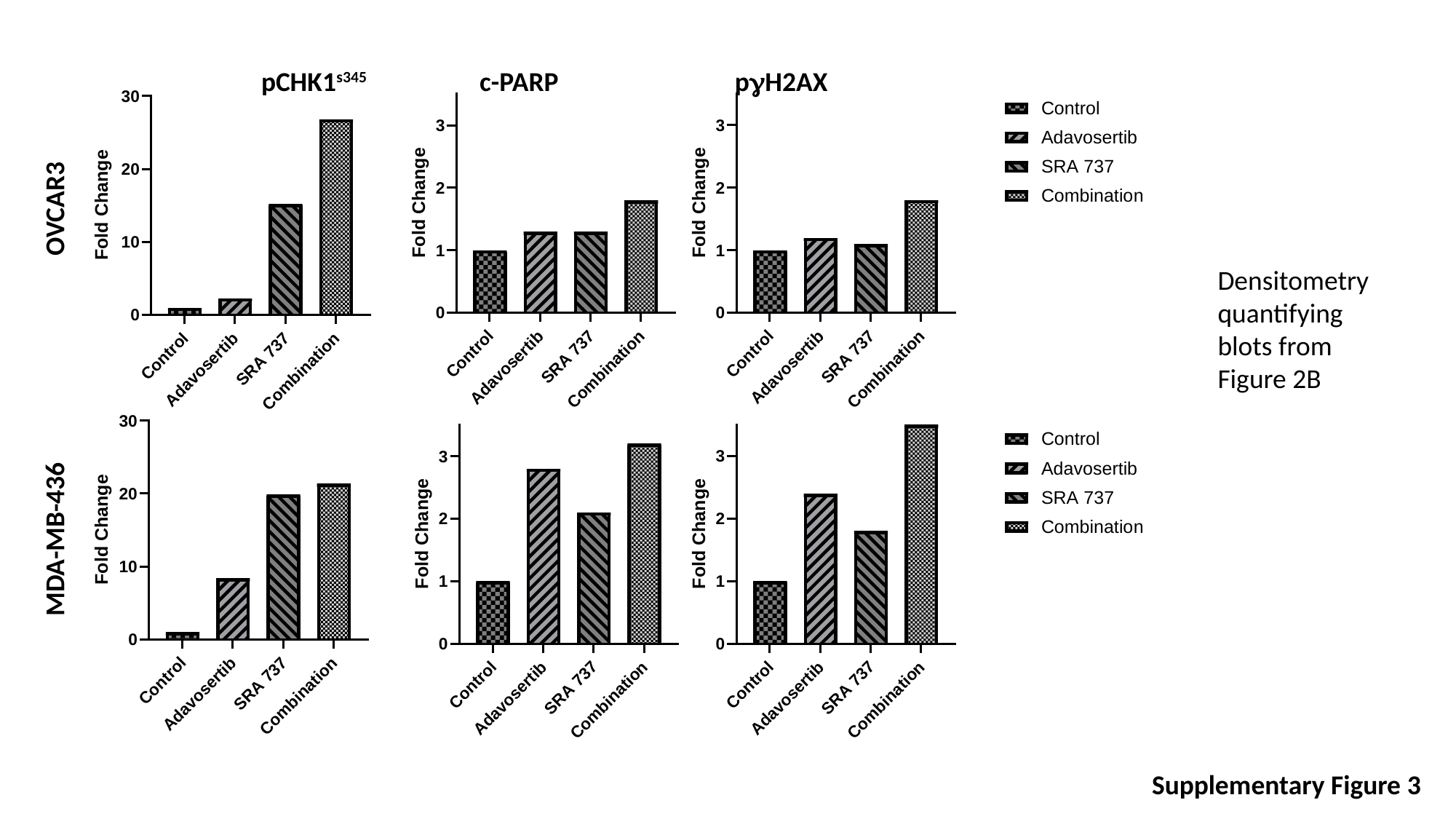

pCHK1s345		c-PARP		 pgH2AX
OVCAR3
Densitometry quantifying blots from Figure 2B
MDA-MB-436
Supplementary Figure 3
